# Burnout in emergency medical and paramedical teams

**DOI:** 10.1192/j.eurpsy.2025.2019

**Published:** 2025-08-26

**Authors:** M. Trabelsi Ajili, M. Jemai, B. Hattay, S. Othmani, H. Hedhli, S. Jouini

**Affiliations:** 1 Emergency, Charles Nicolle’s Hospital; 2 Emergency, Hospital of Charles Nicolle, Tunis, Tunisia

## Abstract

**Introduction:**

Burnout is defined by the WHO as “a feeling of intense fatigue, loss of control and inability to achieve concrete results at work”. Among the most affected populations, are medical and paramedical teams, particularly those exposed not only to a sustained work rhythm but also to frequent confrontations with suffering and death, such as emergency staff.

**Objectives:**

Evaluate the frequency of burnout among medical and paramedical staff and determine the factors associated with it.

**Methods:**

A 1-month cross-sectional study (November to December 2023) was conducted in the emergency department of Charles Nicolle’s hospital, Tunis. We included all medical and paramedical staff. Data were collected using an anonymous online questionnaire on Google Forms. We used the Maslach Burnout Inventory (MBI) as a rating scale.

**Results:**

Forty-five forms were completed. The average age was 29 ±2 years, with a male predominance (69%). Twenty-four percent were already being treated for a psychiatric condition. The average length of service in the emergency department was 8 ± 2 years.

The prevalence of burnout was 98%. Burnout was high in 43% of study participants. Fifty-two percent had a high level of burnout, 63% had a high level of depersonalization and 88% had a low level of sense of personal accomplishment. High burnout was more common among women (79% vs. 21%). Of the participants with high burnout: 31.6% were family doctors, 21% were nurses; 15.7% were emergency physicians (residents or seniors in emergency medicine) and 15.7% were emergency technicians.

Burnout risk factors were divided schematically into 3 categories: among organizational risk factors, 98% complained of work overload with insufficient human and material resources, 44% reported the absence of listening and support from hierarchy, and 31% experienced the feeling that their missions were imprecise. Among the professional risk factors, 78% reported an incompatibility between salary and workload, 67% did not receive any words of recognition from the patient or his family, and 53% did not have any specific training in stress management. Not being able to take time off as they wished was the most reported personal risk factor (76%).

A desire to improve working conditions was present in 80% of participants.

**Image:**

**Figure:**
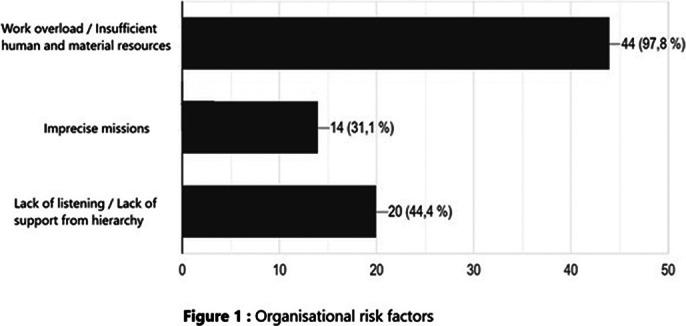

**Image 2:**

**Figure:**
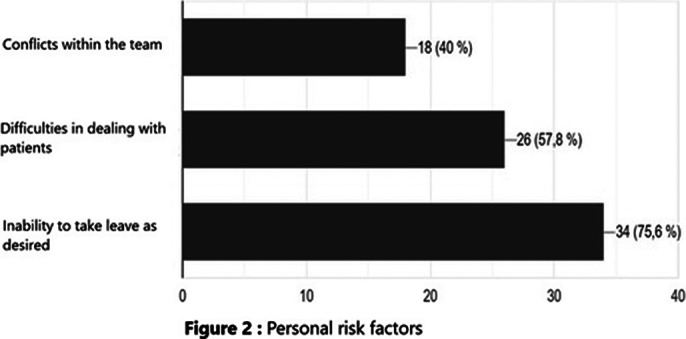

**Image 3:**

**Figure:**
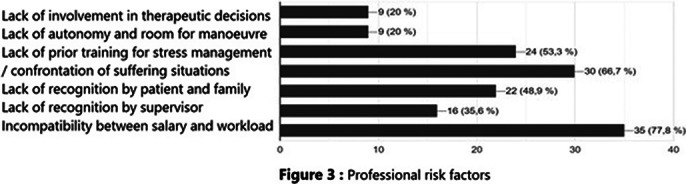

**Conclusions:**

A very high prevalence of burnout has been found within the emergency care team, which could explain their despair of the healthcare system and the increasing exodus rate leading to a potential medical and paramedical desert in Tunisia. It would therefore be urgent to improve working conditions and provide more facilities for young doctors and nurses, particularly in emergency departments.

**Disclosure of Interest:**

None Declared

